# The multiplicity of alternative splicing decisions in *Caenorhabditis elegans* is linked to specific intronic regulatory motifs and minisatellites

**DOI:** 10.1186/1471-2164-15-364

**Published:** 2014-05-14

**Authors:** Dominique A Glauser

**Affiliations:** Department of Biology, University of Fribourg, Chemin du Musée 10, 1700 Fribourg, Switzerland

**Keywords:** Alternate splice sites, Coordination of multiple choices, Regulatory elements, Worm, IMMAD, MASS, SASS

## Abstract

**Background:**

Alternative splicing diversifies the pool of messenger RNA molecules encoded by individual genes. This diversity is particularly high when multiple splicing decisions cause a combinatorial arrangement of several alternate exons. We know very little on how the multiple decisions occurring during the maturation of single transcripts are coordinated and whether specific sequence elements might be involved.

**Results:**

Here, the *Caenorhabditis elegans* genome was surveyed in order to identify sequence elements that might play a specific role in the regulation of multiple splicing decisions. The introns flanking alternate exons in transcripts whose maturation involves multiple alternative splicing decisions were compared to those whose maturation involves a single decision. Fifty-eight penta-, hexa-, and hepta-meric elements, clustered in 17 groups, were significantly over-represented in genes subject to multiple alternative splicing decisions. Most of these motifs relate to known splicing regulatory elements and appear to be well conserved in the related species *Caenorhabditis briggsae*. The usage of specific motifs is not linked to the gene product function, but rather depends on the gene structure, since it is influenced by the distance separating the multiple splicing decision sites. Two of these motifs are part of the CeRep25B minisatellite, which is also over-represented at the vicinity of alternative splicing regions. Most of the remaining motifs are not part of repeated sequence elements, but tend to occur in specific heterologous pairs in genes subject to multiple alternative splicing decisions.

**Conclusions:**

The existence of specific intronic sequence elements linked to multiple alternative splicing decisions is intriguing and suggests that these elements might have some specialized regulatory role during splicing.

**Electronic supplementary material:**

The online version of this article (doi:10.1186/1471-2164-15-364) contains supplementary material, which is available to authorized users.

## Background

The process of splicing determines what part of each gene is included in mature messenger RNA molecules. Alternative splicing allows the generation of more than one transcript isoform from a single gene by the inclusion or exclusion of alternate exons during transcript maturation. Regulated splicing decisions largely depend on nucleotide sequences located in alternate exons or in neighboring introns that recruit specific splicing factors [1–7]. Alternative splicing is a very widespread process among eukaryotes [8]. In human, most multi-exon genes are subject to alternative splicing [9].

**Figure 1:**
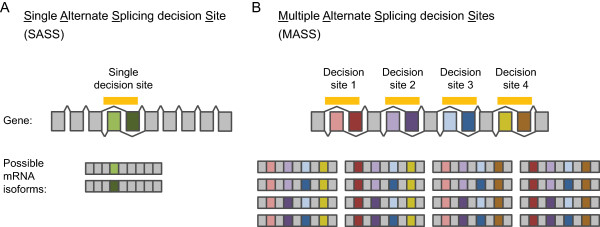
**The combination of multiple alternative splicing decisions diversifies the pool of encoded transcript isoforms.** Scheme comparing Single Alternative Splicing decision Site (SASS) **(A)** and Multiple Alternative Splicing decision Sites (MASS) genes **(B)** and the corresponding possible mature messenger RNA isoforms. It gives an example of the combinatorial complexity resulting from multiple alternative splicing decisions. Constitutive exons are in grey, alternate exons are colored. The case of mutually exclusive alternate exons was chosen to be represented in this figure for the sake of simplicity. However, the definition of MASS and SASS genes used in the present study considers any type of alternative splicing events. By definition, MASS and SASS genes only differ in the number of independent splicing events (see the *Methods* section for details on the MASS and SASS discrimination procedure).

By swapping or skipping portions of transcribed genes, alternative splicing diversifies the repertoire of encoded proteins and its complexity, without requiring an extensive increase in genome size [10, 11]. In genes with a Single Alternative Splicing decision Site (SASS), the number of isoforms is equal to the number of alternate exon definitions (see the illustration in Figure 1A). The number of possible isoforms can be much higher in genes with Multiple Alternative Splicing decision Sites (MASS), due to the combinatorial arrangement of multiple alternate exons (see an example of MASS gene in Figure 1B). The overall physiological significance of the combinatorial complexity generated by alternative splicing is still a matter of debate. Indeed, in most instances, we know relatively little on the consequences of alternative splicing on protein functions, even less when several splicing decisions are combined.

This combinatorial complexity also calls for coordinating multiple splicing decisions. Such coordination mechanisms are particularly important if specific combinations of alternate exons need to be selectively expressed, selectively repressed, or spatially and temporally controlled. Several studies have provided direct or indirect evidence that the pattern of expressed isoforms for given genes might be generated from interdependent splicing decisions [12–15]. For example, a study by Fagnani and collaborators [13] has shown that, in mice, pairs of alternate exons in the same genes could be regulated in a coordinated manner in different tissues. Additionally, Fededa and collaborators [12] identified several genes with nonrandom distributions of mRNA isoforms combining two alternative regions and have suggested the existence of a polar mechanism by which upstream splicing events (in 5’) affect downstream splicing events (in 3’) during transcription.

Recent studies on one MASS gene, the *Caenorhabditis elegans slo-1* BK channel gene (involved in neurotransmission), have provided insights on the nature of intragenic alternative splicing coordination mechanisms and demonstrated their functional significance *in vivo*[14, 16]. Alternative splicing decisions at three sites along *slo-1* produce twelve possible mRNA isoforms: a reasonable complexity that was suitable for systematic and quantitative analyses of expression and function. Three major findings were reported. First, protein domains encoded by distant alternate exons functionally interact to influence the channel biophysical properties [16]. In other words, the impact of several splicing decisions on the protein function is not simply the sum of the impact of each decision; rather, specific isoforms gain unique properties. Thus, there are functional reasons for specific combinations of alternate exons to be selected during the maturation of *slo-1* transcripts. Second, the pattern of isoforms expressed in *C. elegans* cannot be accounted for by independent decisions across the three alternative splicing regions, demonstrating the inter-dependent nature of alternative splicing decisions in *slo-1*. Third, this coordination can be disrupted by a point mutation in a single intronic motif, which not only affects nearby splicing decisions, but also splicing decisions made at distant sites [14]. The disruption of splicing coordination results in physiological impairments, such as dysregulated neurotransmission. These findings highlight the functional significance of intragenic splicing coordination *in vivo* and suggest the existence of specific intronic motifs that are important for coordinating intragenic splicing decisions.

The goal of the present study was to identify, at a genomic scale, intronic motifs that may specifically regulate multiple splicing decisions in *C. elegans*. Similarly to a study investigating splicing *cis-*regulatory motifs across *Caenorhabditis* species [17], the frequencies of pentameric, hexameric, and heptameric sequence elements were compared between two groups of introns flanking alternate exons: introns from MASS and SASS genes. MASS and SASS genes only differ in the number of independent splicing events (see an illustration of their definition in Figure 1). If mechanisms that are specific to multiple alternative splicing decisions (like splicing coordination) are very uncommon or do not rely on sequences located in introns flanking alternate exons, then the sequence composition in the SASS and MASS groups should be similar. Conversely, if those mechanisms are more prevalent, then the sequence composition should diverge between the two groups. In this case, motifs that are more frequent in the MASS group represent motifs with a potential regulatory role specific to the multiplicity of splicing decisions.

The results of the present study indicate that the sequence composition of introns at the vicinity of alternate exons is indeed different whether only one or several alternative splicing decisions are engaged. Motifs enriched in the MASS group were called IMMADs, for Intronic Motifs linked to Multiple Alternative splicing Decisions. Most IMMADs appear to be well conserved in the related species *Caenorhabditis briggsae*. IMMADs include several oligomers with known splicing regulation functions and one minisatellite (CeRep25B). In conclusion, this study suggests that several intronic *cis-*regulatory elements have a specific regulatory role associated with multiple alternative splicing decisions along single transcripts.

## Results

### Identification of Intronic Motifs linked to Multiple Alternative splicing Decisions (IMMADs)

A set of 2322 alternatively spliced genes, retrieved from WormBase (WS235 [18]) was classified according to the number of sites where splicing decisions occur (see *Methods*). This dataset included a total of 752 MASS genes and 1570 SASS genes. The analysis of the differential sequence composition between the introns flanking alternate exons of MASS and SASS genes identified a total of 63 oligomers (22 pentamers, 22 hexamers, and 19 heptamers) clustered in 18 different motifs that are significantly enriched in the MASS group (*p* < 1E-5, Additional file 1). 644 out of the 752 MASS genes (86%) harbored at least one of these IMMADs in introns flanking alternate exons.

**Figure 2:**
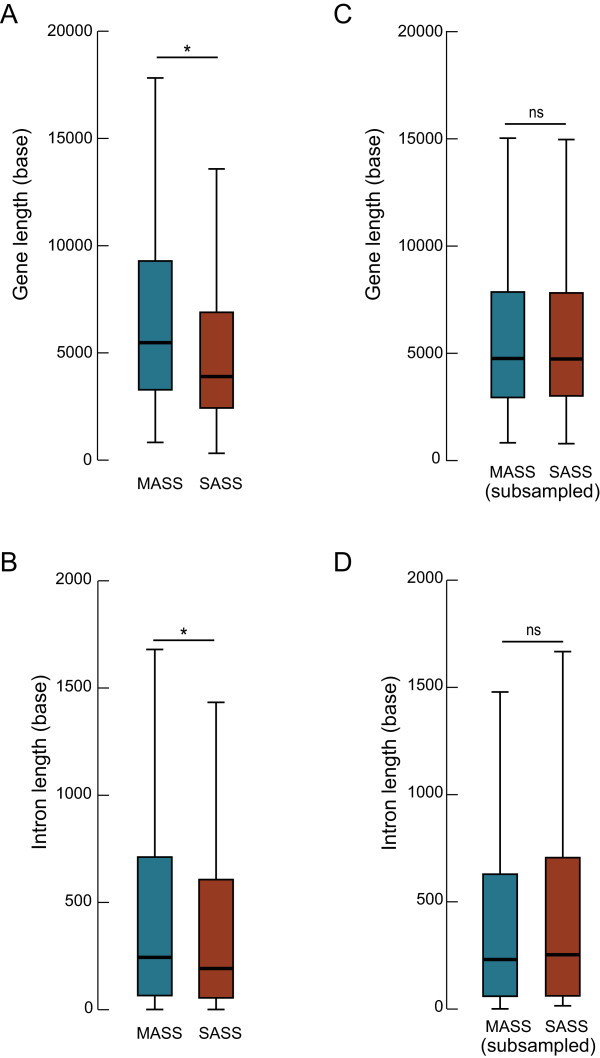
**Comparison of gene and intron lengths between SASS and MASS genes. A)** Gene lengths in the full sample of SASS and MASS genes. A significant difference was found by Mann Whitney U test (*p < .001). **B)** The length of introns flanking alternate exons in the full sample of SASS and MASS genes. A significant difference was found by Mann Whitney U test (*p < .001). **C)** Gene lengths in the stratified subsample of SASS and MASS genes. No significant difference was found by Mann Whitney U test (ns, *p =* .86). **D)** The length of introns flanking alternate exons in the stratified subsample of SASS and MASS genes. No significant difference was found by Mann Whitney U test (ns, *p =* .19).

The presence of motifs enriched in the MASS group could indicate their implication in splicing regulation or, alternatively, reflect structural or functional differences between the MASS and SASS genes. To control for these potential confounding effects, a more extensive comparative analysis of the MASS and SASS genes was performed. SASS and MASS genes had identical nucleotide composition in introns flanking alternate exons, their chromosomal distribution was similar, and a Gene Ontology (GO) term analysis showed no gene product function difference between the two groups. However, the size of the genes and the size of the introns flanking alternate exons were larger in the MASS group (Figure 2A and B). A stratified subsampling of the MASS and SASS genes was therefore performed in order to match the two length distributions in both groups (Figure 2C and D). When reiterated with the subsampled groups, the oligomeric motif analysis re-identified 58 out of the initial 63 elements (*p* < 1E-2, see Additional file 2), corresponding to 17 out of 18 initially identified IMMADs. The oligomers corresponding to the CACACAC motif did not pass this size-subsampling control analysis and this motif was excluded from the subsequent analyses. Table 1 reports the 17 IMMADs that passed the size-subsampling control analysis and for which a confounding effect of systematic structural differences between SASS and MASS genes can be ruled out. These motifs might have a specific role associated with the multiplicity of alternative splicing decisions and have been further analyzed.

**Table 1:** **Hepta-, hexa-, and pentameric Intronic Motifs linked to Multiple Alternative splicing Decisions (IMMADs)**

Motif	MASS/SASS ratio	Corrected ***p***-value	Number of MASS genes
GGTCTGC	4.0	7.9E-13	24
AGCAGAC	4.0	7.9E-13	36
CAHCC	3.5	8.4E-22	454
CCACA	2.8	1.9E-17	359
RAGAAG	2.7	2.4E-15	339
AGCCTCA	2.4	1.0E-12	38
CCATCGT	2.4	2.1E-07	52
ACATTCG	2.2	3.3E-06	57
TCTCTCT	2.1	4.0E-39	118
WCTTCTT	2.0	1.2E-12	227
GAATGTT	1.9	5.0E-12	119
GATGAC	1.8	1.3E-11	142
ACYCCA	1.7	5.2E-11	163
GTCGT	1.7	4.0E-11	299
CCAGC	1.5	4.0E-15	286
TGGAC	1.3	1.4E-07	275
AGGAG	1.3	8.4E-10	301

Seventeen motif groups are significantly enriched in the introns flanking alternate exons among the MASS genes as compared to the SASS genes (*p* < 1E-5, by Fisher’s exact tests with Bonferroni corrections). IUPAC ambiguity codes were used: R = A or G; W = A or T; H = A, C, or T; Y = C or T.

### IMMADs are globally over-represented in introns flanking alternate exons in MASS genes

The method reported here-above to identify IMMADs compared the frequencies of oligomeric motifs among intronic sequences in MASS and SASS groups. In principle, IMMADs could have been identified because they are globally over-represented across MASS genes, or because they are present with an extreme number of copies in only few MASS genes. To control for the number of IMMAD repeats, the frequencies of the genes harboring at least one IMMAD copy in the MASS genes were compared to those in the SASS genes. This approach is not influenced by the number of repeats within each gene. This analysis showed a significantly higher gene frequency in the MASS versus SASS groups for each of the 17 identified IMMADs (*p* < *.*01, Additional file 3). These results indicate that the IMMADs are globally over-represented in introns flanking alternate exons in MASS genes and that their enrichment is not solely contributed by very few genes with multiple IMMAD repeats.

### IMMAD conservation in Caenorhabditis briggsae

**Figure 3:**
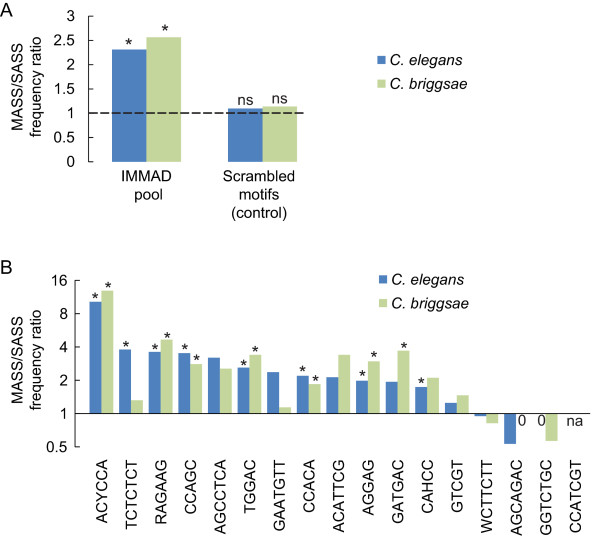
**Conservation of IMMADs in** ***C. briggsae*** . The frequencies of IMMADs in introns flanking alternate exons were compared between *C. elegans* MASS and SASS genes, as well as between groups of orthologous genes in *C. briggsae*. These analyses focused on genes with a conserved exon-intron structure and for which the definition of *C. briggsae* introns of interest was unambiguous (38 MASS and 187 SASS genes, see *Methods* for more details). **A)** General analyses with a motif pool including the 17 IMMADs initially identified with a larger sample of MASS and SASS genes in *C. elegans* (Table 1). As control, the frequency of a population of scrambled IMMADs was compared across the MASS and SASS genes in both species. MASS/SASS frequency ratios are reported. Fisher’s exact tests were performed to evaluate the IMMAD enrichment in the MASS group versus the SASS group. *p < .01 (indicating a ratio significantly different from one). ns, not significant. **B)** Separate analyses for each IMMAD. MASS/SASS frequency ratios for each IMMAD are reported. Note the log scale on the vertical axis. Fisher’s exact tests were performed to evaluate the specific IMMAD enrichments in the MASS group versus the SASS group. A Bonferroni correction for multiple comparisons was applied. *p < .01 (indicating a ratio significantly different from one). *p*-values are reported in Additional file 4. na, not applicable because there was no occurrence in the SASS group.

The evolutionary conservation of IMMADs was assessed in the related species *C. briggsae* by comparing genes orthologous to *C. elegans* MASS and SASS genes. The *C. briggsae* sequences of introns flanking exons that are orthologous to alternate exons in *C. elegans* were defined and analyzed. This analysis was complicated by the fact that the exon-intron structure of most genes is not conserved between the two species, which diverged about 100 million years ago [19]. Therefore, the definition of the orthologous introns of interest in *C. briggsae* was restricted to introns in genes whose exon-intron structure is conserved across the two species [20]. This corresponded to 223 alternatively spliced genes (36 MASS and 187 SASS genes), a markedly smaller sample than for the initial MASS/SASS comparison in *C. elegans*. In this specific subsample of *C. elegans* MASS and SASS genes, the overall frequency of IMMADs was still significantly higher in the MASS as compared to the SASS group (fold change: 2.31; *p =* 5.95E-28, by Fisher’s exact test, Figure 3A and Additional file 4). This enrichment was also found within *C. briggsae* sequences (fold change: 2.56; *p =* 7.12E-42, by Fisher’s exact test, Figure 3A and Additional file 4). A control set of scrambled IMMAD sequences was enriched neither in the *C. elegans* nor in the *C. briggsae* MASS sequences (Figure 3A)*.* These results indicate that, when analyzed as a whole, the pool of IMMADs identified in the initial MASS/SASS comparison in *C. elegans* is (a) still enriched in the *C. elegans* subset of MASS genes with conserved exon-intron structures and (b) also enriched in the corresponding *C. briggsae* orthologs.

Next, separate analyses for each IMMAD were conducted in order to determine if some IMMADs might be more conserved than others. In the *C. elegans* subsample, 13 out of 17 IMMADs had still a MASS/SASS frequency ratio greater than one (range: 1.25-14.93, Figure 3B). Strikingly, these exact same 13 IMMADs had also a MASS/SASS frequency ratio greater than one in *C. briggsae* (range: 1.13-12.9, Figure 3B), even if not all enrichments reached the *p*-value threshold set for statistical significance. Collectively, these results indicate that the specific enrichment of IMMAD sequences in *C. elegans* MASS genes is, for most of them, conserved in the orthologous genes of *C. briggsae*.

### Comparison with known splicing regulatory elements

In order to determine whether the IMMADs might serve as Splicing Regulatory Elements (SREs), their list was compared to published lists of SREs in *C. elegans* and other species [17, 21–24]. An overlap was observed for members of most IMMAD groups (13 out of 17, Table 2). Nine IMMAD groups included intronic splicing elements reported by Kabat and collaborators in *C. elegans*[17]. This result corresponds to a significant over-representation of the previously detected *C. elegans* intronic elements within the IMMADs identified here (*p* = 2.58E-4 by Fisher’s exact test, see *Methods*). Four motifs had also been identified as conserved intronic splicing elements across humans, dogs, mice, and rats [21]. This finding is consistent with a large conservation of the SRE sequences across species [17]. In addition, five motifs were related to exonic splicing elements identified in mammals [22–24]. Those motifs might work both as intronic and exonic splicing elements, a property often observed in mammals [4]. Collectively, this comparative analysis points to 13 SREs that might have some degree of specialization in splicing coordination or might participate in other unknown functions associated with the need for multiple intragenic splicing decisions.

**Table 2:** **Comparison of hepta-, hexa-, and pentameric IMMADs with previously reported Splicing Regulatory Elements (SREs)**

Motif	Overlap with previously published SREs				
	Kabat ***et al.*** [17]	Yeo ***et al.*** [21]	Ke ***et al.*** [22]	Fairbrother ***et al.*** [23]	Goren ***et al.*** [24]
AGCAGAC	-	-	yes	-	-
GGTCTGC	yes	-	-	-	-
CCATCGT	-	-	-	-	-
ACATTCG	-	-	-	-	-
AGCCTCA	-	-	-	-	-
GAATGTT	-	-	-	-	-
RAGAAG	-	-	yes	yes	yes
WCTTCTT	yes	yes	yes	-	-
ACYCCA	yes	-	yes	-	-
GATGAC	-	-	yes	-	yes
TCTCTCT	yes	yes	-	-	-
CCAGC	-	yes	-	-	-
TGGAC	yes	-	-	-	-
CAHCC	yes	-	-	-	-
GTCGT	yes	-	-	-	-
AGGAG	yes	-	-	-	-
CCACA	yes	yes	-	-	-

On the other hand, there were four motifs for which no evidence for a splicing regulatory role was found in the literature. Those might represent elements either lacking regulatory functions or with a specific role in the regulation of multiple splicing decisions that was not apparent in previous analyses. Further investigations will be required to clarify this issue.

### Comparison with known RNA-binding protein recognition motifs

In order to determine whether IMMADs might be bound by RNA-binding proteins, IMMAD sequences were compared to RNA-binding protein recognition sequences reported in the literature. A recently published study by Ray and collaborators [25] used the RNAcompete method [26] to map the binding motifs of more than 200 RNA-binding proteins across several species and integrated these new data with a review of the literature on the motifs recognized by RNA-binding proteins. Four IMMADs were found to relate to these RNA-binding protein recognition motifs (Table 3). *C. elegans* homologs for the four corresponding RNA-binding proteins were identified using BLAST searches (BLASTN 2.2.28 [27]) (Table 3). Because the sequence specificities of RNA-binding proteins are strongly evolutionary conserved [25], these results suggest that at least some of the identified IMMADs could be targeted by RNA-binding proteins.

**Table 3:** **IMMADs similar to previously reported motifs recognized by RNA-binding proteins**

Motif	RNA-binding protein	Species	Reference	***C. elegans*** homolog
WCTTCTT	PTB1	*H. sapiens*	[25, 28]	PTB-1
CAACC	HNRNPK	*H. sapiens*	[25, 29]	PES-4
AGGAG	SRSF2	*H. sapiens*	[25, 26]	RSP-4
RAGAAG	SRSF10	*H. sapiens*	[25, 30]	RSP-4/RSP-6

### Ontology of genes harboring specific IMMADs

In order to address whether the IMMADs identified here are associated with genes sharing similar functions, GO term analyses were performed. First, the MASS group of genes was compared to the rest of the genome. Several *Biological Process* GO terms were significantly enriched in the MASS group (Table 4, and Additional file 5), including locomotion and development. The same GO terms were identified when comparing the SASS genes to the whole genome. This is consistent with the previous analysis showing no GO difference between MASS and SASS genes (see above). These results highlight that alternative splicing in *C. elegans* is more common in groups of genes involved in specific functions, which is reminiscent of observations made in mammals [13].

**Table 4:** **Gene Ontology (GO) analysis: most significantly enriched GO terms in MASS genes as compared to the whole genome**

GO Term	Description	***p***-value	FDR ***q*** value	Enrichment
GO:0065007	biological regulation	3.35E-28	1.13E-24	1.82
GO:0044699	single-organism process	8.98E-28	1.52E-24	1.5
GO:0050789	regulation of biological process	1.01E-27	1.15E-24	1.83
GO:0008150	biological_process	1.04E-24	8.78E-22	1.29
GO:0009987	cellular process	4.41E-24	2.99E-21	1.57
GO:0044763	single-organism cellular process	2.92E-22	1.65E-19	1.74
GO:0032502	developmental process	3.95E-21	1.91E-18	1.69
GO:0050794	regulation of cellular process	2.12E-18	8.97E-16	1.99
GO:0048518	positive regulation of biological process	2.52E-18	9.5E-16	2.06
GO:0044767	single-organism developmental process	2.6E-18	8.8E-16	1.68
GO:0048856	anatomical structure development	2.65E-17	8.15E-15	1.71
GO:0009791	post-embryonic development	2.47E-16	6.97E-14	2.01
GO:0002119	nematode larval development	8.04E-16	2.09E-13	1.99
GO:0002164	larval development	8.45E-16	2.04E-13	1.99
GO:0040011	locomotion	1.76E-15	3.98E-13	2.11
GO:0040008	regulation of growth	3.78E-15	8E-13	2.02
GO:0040012	regulation of locomotion	1.21E-14	2.42E-12	4.14
GO:0048519	negative regulation of biological process	1.68E-14	3.17E-12	2.82
GO:0016043	cellular component organization	3.67E-14	6.55E-12	2.41
GO:0007610	behavior	5.04E-14	8.54E-12	3.13

Top 20 GO terms ranked according to the *p*-values for enrichment in the MASS group of genes, as compared to the *C. elegans* whole genome annotations. FDR, False Discovery Rate.

Second, subgroups of MASS genes containing each of the specific IMMADs in introns flanking alternate exons were compared to the whole genome. In most instances, the predominant GO terms were the same as those found in the general comparison of MASS genes with the rest of the genome (Additional files 5 and 6). This suggests that the functions of the different IMMAD-harboring gene subsets might not strongly diverge as compared to those of other alternatively spliced genes. To confirm this observation, the same subgroups of MASS genes containing each of the specific IMMADs were compared to the pool of alternatively spliced genes (MASS and SASS together). For all but one motif, there was no significant enrichment (with a *q* value threshold at .01, Additional file 6).

Collectively, these results show that the presence of specific IMMADs in introns flanking alternate exons are not generally associated with specific gene functions.

### Specific IMMADs are associated with the need for coordination over long or short distances

**Figure 4:**
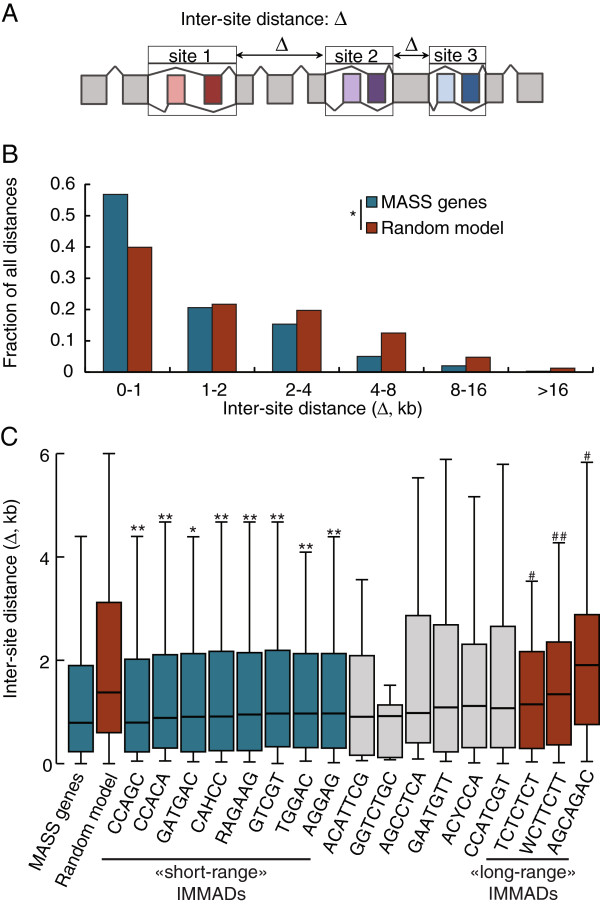
**Analysis of the distances between multiple splicing decision sites. A)** Definition of the inter-site distance: Δ. **B)** The observed distribution of inter-site distances within MASS genes was compared to a simulated distribution based on a model picking random inter-site distances in a simulated pool of 6510 transcripts. The total gene length and intron length distributions in the simulated pool were the same as the ones in the MASS genes (*p < .001 by Mann Whitney U tests). **C)** Inter-site distance distributions for subsets of MASS genes harboring specific IMMADs were compared to the random model and to the full MASS gene interval distributions. A Kruskal-Wallis test indicated a significant gene group effect (*p* < .001). Mann-Whitney U tests were performed to compare each IMMAD-specific group to the random model and to the MASS gene group, respectively. **p < .01; *p < .05 versus random model; ##, *p* < .01; #, *p* < .05 versus the MASS genes. IMMAD groups depicted in grey displayed no significant differences with either control groups.

Among the MASS genes, the distance between separate sites where alternative splicing decisions occur (Figure 4A) covers a wide range, from less than 100 bases to up to 20 kb. However, the inter-site distances are significantly shorter than in a model randomly picking inter-site distances in a simulated pool of 6510 transcripts, matching the intron size and total length of MASS genes (*p* < .001 by Mann Whitney U test, Figure 4B). In other words, pairs of introns flanking alternate exons implicated in multiple splicing decisions are located closer than are pairs of introns taken randomly. This suggests that potential splicing coordination mechanisms might tend to work over regions of limited size. To evaluate if specific IMMADs might be preferentially used for long-range or short-range splicing coordination, the distributions of inter-site distances were computed for the gene subgroups harboring each of the 17 IMMADs. These distributions were compared to two controls: the distribution from the random model and the distribution observed in the MASS genes. A Kruskal-Wallis test indicated significant differences across these groups (*p* < .001) and was followed by Mann-Whitney U tests (corrected for multiple comparisons) to decipher individual differences between each IMMAD-harboring group and the two controls. Results show that eight groups harboring specific IMMADs are significantly shorter than the random model (Figure 4C). These IMMADs might preferentially act over short distances. On the other hand, three groups were not different from the random model, but significantly longer than the MASS gene group. These IMMADs might tend to act over longer distances. Collectively, these results suggest that some IMMADs might work preferentially for long distance coordination processes, while others might work preferentially over shorter distances.

### Two heptameric IMMADs are part of the CeRep25B minisatellite repeat

It was intriguing that the two top hit heptamers in our IMMAD analysis (Table 1) were nearly a perfect reverse complement of each other: AGCAGAC and GGTCTGC (6 base match). In order to examine if they could be part of a larger palindromic element, the co-localization of these motifs was thus examined in introns of MASS genes. The two motifs were found together much more frequently in the same intron than one would have expected by chance based on their individual frequencies (significant co-occurrence enrichment: *p* < .01, by Fisher’s exact test). In most genes in which these motifs co-occur (6/8), the two oligomers were found in stretches of repeated palindromic (or nearly-palindromic) sequences corresponding to the previously characterized CeRep25B minisatellite [31]. This minisatellite consists of 24 bases repeated several times. The genomic distribution of this minisatellite is essentially restrained to specific clusters along chromosome III and, to a lesser extent, chromosome II [31]. 75% of these repeats lie in introns and an undetermined portion of the remaining repeats might also be part of nascent transcript 5’UTRs, which are poorly characterized in *C. elegans* due to trans-splicing [32]. The occurrence of CeRep25B minisatellites was then examined within introns flanking alternate exons in MASS and SASS genes. A significant enrichment of this satellite was found in the MASS group (*p* = .0025 by Fisher’s exact test). Actually, this minisatellite was never found in the introns flanking alternate exons in the SASS group. Together with the fact that this minisatellite contains repeats of two previously identified SREs [17, 22], this finding raises the possibility that this minisatellite influences splicing, as shown previously in other organisms for similar repeated sequences [33].

In order to determine if the CeRep25B minisatellite was solely responsible for the identification of the AGCAGAC and GGTCTGC motifs during the initial IMMAD screen, their enrichment within the MASS genes as compared to the SASS genes was recalculated while excluding the CeRep25B-containing genes from the analysis. For the GGTCTGC motif, the enrichment in the MASS group was no longer statistically significant (fold change = 1.71; *p* = .03 by Fisher’s exact test). This result indicates that the GGTCTGC motif might only be enriched within the MASS group because it is part of the MASS-associated minisatellite CeRep25B. In contrast, the AGCAGAC motif was still significantly enriched in the MASS group upon removal of the CeRep25B containing genes (fold change = 2.88; *p* = 5.24E-7 by Fisher’s exact test). This indicates that the AGCAGAC heptamer is enriched within the introns flanking alternate exons in MASS genes, both as part of the CeRep25B minisatellite and when occurring outside of it.

### Additional oligomeric IMMADs are part of larger sequence elements

**Figure 5:**
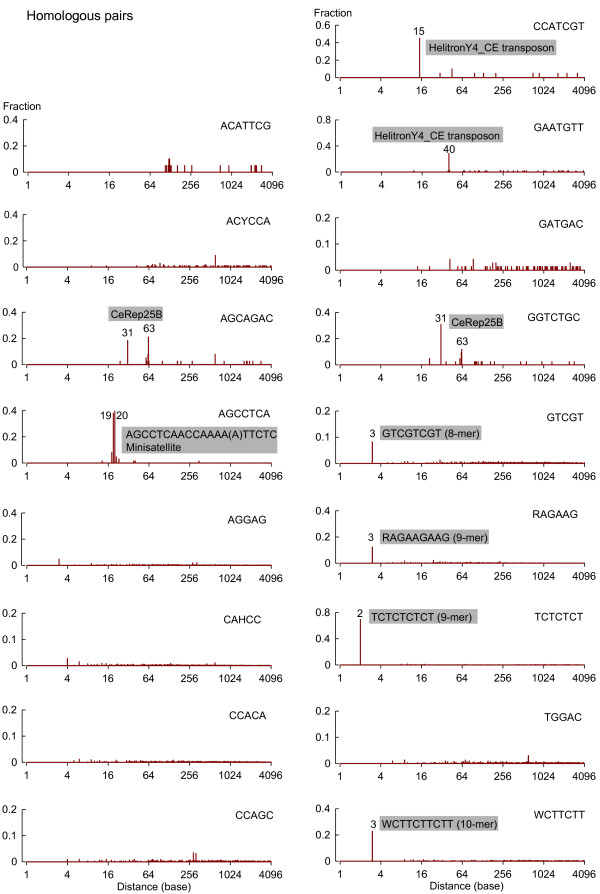
**Distance distributions between homologous pairs of IMMADs.** IMMAD coordinates within introns flanking alternate exons of the MASS genes were computed to determine the distances between homologous pairs of consecutive IMMADs. The inter-motif distance distributions are reported for the 17 IMMAD groups shown in Table 1.

One could wonder whether other penta-, hexa-, or heptameric IMMADs identified in the MASS-SASS comparison (Table 1) could also be part of repeated elements. To address this question and identify putative larger repeated elements, the distance separating homologous pairs of IMMADs was examined in the introns flanking alternate exons of MASS genes. The distribution of distances between consecutive motifs was plotted for each of the 17 IMMADs (Figure 5). The presence of a given motif in repeated sequences is indicated by prominent peaks along the distribution. For example, the plots for the AGCAGAC and GGTCTGC motifs both clearly highlight two peaks (at 31 and 63 bases), corresponding to the most common repeated intervals within the CeRep25B minisatellite. Similarly, two peaks (at 19 and 20 bases) for the AGCCTCA motif, one peak (at 15 bases) for the CCATCGT motif, and one peak (at 40 bases) for the GAATGTT motif were predominant. These represented ~80%, ~45%, and ~29% of the total intervals, respectively, and signaled the presence of larger repeated elements, which include these motifs. These were analyzed in more details (see the next two paragraphs).

The AGCCTCA motif was part of a tandem repeat minisatellite comprising 20 or 19 base pairs. This minisatellite was found in 3 MASS genes (Y38C1AA.1, F29C4.7, and ZK57.4c). This minisatellite was not reported in the Repbase Update database (6-22-2013) [34]. Within these three MASS genes the repeats had the consensus sequence AGCCTCAACCAAAA(A)TTCTC. No occurrence of this minisatellite was found within the SASS genes. However, because of the very few genes involved, it is not possible to conclude on an association between this minisatellite and the multiplicity of alternative splicing decisions. When removing this minisatellite to re-perform the sequence comparison between introns flanking alternate exons in MASS and SASS genes, the AGCCTCA motif was not significantly over-represented in the MASS genes anymore. Thus, it is possible that the initial identification of the AGCCTCA motif was an artifact due to the exceptional weight conferred by only a few genes harboring several repeats.

The CCATCGT motif was part of a tandem repeat of 15 bases with the sequence CCATCGTGG(T/C)GAGAC, which is part of a transposon from the HelitronY4_CE family [34, 35]. The GAATGTT motif was also part of the same transposon, but was present in a different tandem repeat (40 bases) with the consensus sequence: AAAATTCTGGAATGTTCCAGAACTTTCTAGAAAAATTGGG. Among MASS genes, this transposon was present only in the R11A5.4 gene. No occurrence of this transposon was found within the SASS genes. However, because only one MASS gene is involved, it is not possible to conclude on a potential association between this transposon and the multiplicity of alternative splicing decisions. When removing this transposon to re-perform the sequence comparison between introns flanking alternate exons in MASS and SASS genes, the CCATCGT and GAATGTT motifs were both still significantly enriched in the MASS genes (*p* = 2.47E-4 and 2.59E-4, respectively, by Fisher’s exact tests). This means that, regardless of their inclusion as repeated motifs in the HelitronY4_CE transposon, the CCATCGT and GAATGTT heptamers are over-represented in the introns flanking alternate exons of MASS genes.

In addition, peaks for shorter inter-motif distances (at 1-3 bases) were found for the GTCGT, RAGAAG, TCTCTCT, and WCTTCTT motifs (Figure 5). These results point to the existence of octa-, nona-, and deca-meric elements including two repetitions of these shorter oligomers. Among the four longer oligomers, two were significantly over-represented in the introns flanking alternate exons from the MASS genes as compared to the SASS genes: RAGAAGAAG (fold change = 2.0; *p* = 1.07E-5), and TCTCTCTCT (fold change = 2.6; *p* = 2.60E-8).

Collectively, the results of the analysis of the distance separating homologous IMMADs show that several of them occur as part of larger elements such as minisatellites or longer oligomers.

### Some oligomeric IMMADs preferentially occur in specific pairs

**Figure 6:**
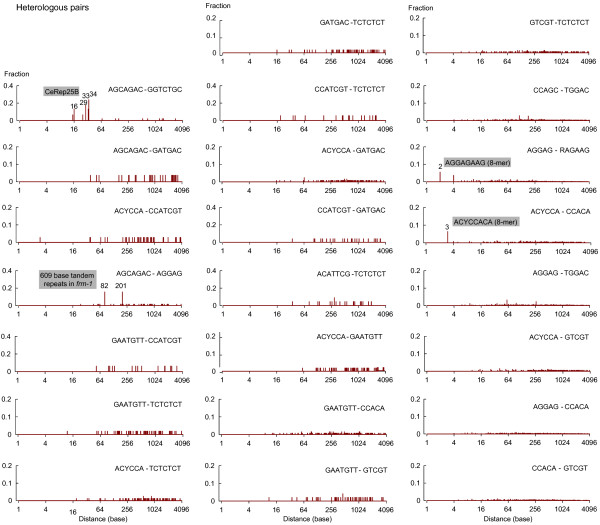
**Distance distributions between heterologous pairs of IMMADs.** IMMAD coordinates within introns flanking alternate exons of the MASS genes were computed to determine the distances between heterologous pairs of consecutive IMMADs. The inter-motif distance distributions are reported for the 23 IMMAD heterologous pairs showing significant co-occurrence enrichments (Table 5).

One could wonder whether some IMMAD types tend to occur together, which might indicate they are recurrently involved in joint regulation. To address this question, a systematic co-occurrence analysis was performed for every of the 136 possible heterologous pairs of the 17 oligomeric IMMADs identified in the present study. At least one motif pair was found in the majority of MASS genes (406/752). Based on the frequencies of individual IMMADs, this does not however constitute a general over-representation of IMMAD pairs. Each of the 136 specific pairs was then systematically tested for co-occurrence enrichment. Twenty-three pairs had a significant co-occurrence enrichment (*p* < .01 by Fisher’s exact tests, corrected for multiple comparisons; odds ratio: 17.6 – 2.2, see Table 5). The top hit was the AGCAGAC-GGTCTGC pair, found in the CeRep25B minisatellite. In order to determine whether the remaining oligomer pairs were also part of larger elements, their relative positions were computed and the distribution of the distances between consecutive elements was plotted (Figure 6). Prominent peaks were observed for four heterologous oligomeric IMMAD pairs:

**Table 5:** **Co-occurrence analysis of IMMAD heterologous pairs**

IMMAD pair	Odds ratio	Number of genes	***p***-value*
AGCAGAC-GGTCTGC	17.6	8	1.2E-04
AGCAGAC-GATGAC	5.1	15	5.2E-03
ACYCCA-CCATCGT	5.0	23	1.3E-04
AGCAGAC-AGGAG	4.9	20	3.5E-03
GAATGTT-CCATCGT	4.8	19	7.6E-04
GAATGTT-TCTCTCT	4.8	39	8.1E-08
ACYCCA-TCTCTCT	4.5	46	4.2E-08
GATGAC-TCTCTCT	4.3	39	8.2E-07
CCATCGT-TCTCTCT	4.2	17	6.6E-03
ACYCCA-GATGAC	4.0	49	1.3E-07
CCATCGT-GATGAC	3.9	18	9.3E-03
ACATTCG-TCTCTCT	3.9	18	8.9E-03
ACYCCA-GAATGTT	3.6	47	3.9E-06
GAATGTT-CCACA	3.6	67	6.7E-07
GAATGTT-GATGAC	3.0	35	9.4E-04
GTCGT-TCTCTCT	3.0	46	2.1E-04
CCAGC-TGGAC	3.0	65	7.0E-06
AGGAG-RAGAAG	2.8	74	8.9E-06
ACYCCA-CCACA	2.5	76	2.3E-04
AGGAG-TGGAC	2.4	65	8.0E-04
ACYCCA-GTCGT	2.4	55	3.6E-03
AGGAG-CCACA	2.3	98	3.3E-04
CCACA-GTCGT	2.2	89	1.0E-03

23 out of the 136 possible IMMAD heterologous pairs present a co-occurrence enrichment within the introns flanking alternate exons of MASS genes. The reported *number of genes* is the number of MASS genes where a given IMMAD pair occurs. *the co-occurrence enrichment *p*-values were calculated by Fisher’s exact tests with Bonferroni corrections for multiple comparisons.

First, the AGCAGAC-GGTCTGC pair, found in the CeRep25B element, displayed four peaks in its distribution (at 16, 29, 33, and 34 bases), corresponding to the most common intervals between the two oligomers within this minisatellite (Figure 6).

Second, the AGCAGAC-AGGAG distance distribution displayed two peaks (at 81 and 201 bases), each contributing to nearly 16% of the inter-oligomer distances (Figure 6). Both peaks were due to the presence of a sequence stretch containing 10 tandem repeats of 609 bases in only one gene (*frm-1*). A BLAST search (BLASTN 2.2.28 [27]) for this DNA sequence revealed no other occurrence in the *C. elegans* genome. Because the co-occurrence analysis (reported in Table 5) is based on gene counts rather than on motif pair counts (see *Methods*), the presence of this repeated sequence in a single gene is unlikely to have yielded the “artifactual” identification of the AGCAGAC-AGGAG pair. This conclusion was confirmed by the result of an analysis excluding the *frm-1* gene to recalculate the co-occurrence enrichment of the AGCAGAC-AGGAG pair (*p* = 9.0E-5, by Fisher’s exact test).

Third, the AGGAG-RAGAAG distance distribution displayed one peak (at 2 bases), corresponding to the AGGAGAAG octamer (Figure 6). The AGGAGAAG octamer accounted only for 7% of the total AGGAG-RAGAAG pairs. However, this octamer was significantly enriched in the MASS as compared to the SASS group of introns flanking alternate exons (fold change = 2.03; *p* = 2.2E-3 by Fisher’s exact test). The co-occurrence analysis excluding this octamer still yielded a largely significant co-occurrence enrichment for the RAGAAG-AGGAG motif pair (*p* = 1.16E-6 by Fisher’s exact test). Collectively, these data indicate that both the AGGAGAAG octamers and pairs of distant RAGAAG and AGGAG motifs are associated with multiple splicing decisions.

Fourth, the ACYCCA-CCACA distance distribution displayed one peak (3 bases), corresponding to the ACYCCACA octamer (Figure 6). The ACYCCACA octamer accounted only for 6% of the total ACYCCACCACA pairs. However, this octamer was significantly enriched in the MASS as compared to the SASS group of introns flanking alternate exons (fold change = 2.89; *p* = 2.80E-5 by Fisher’s exact test). The co-occurrence analysis excluding this octamer still yielded a significant co-occurrence enrichment for the ACYCCA-CCACA motif pair (*p =* 2.03E-5 by Fisher’s exact test). Collectively, these data indicate that both the ACYCCACA octamers and pairs of distant ACYCCA and CCACA motifs are associated with multiple splicing decisions.

Apart from the four cases detailed above, there was no predominant distance peak in the remaining 19 pairs of oligomeric IMMADs with enriched co-occurrence (Figure 6). Thus, contrary to what happens in the CeRep25B minisatellite and when they are part of larger oligomers, most co-occurring penta-, hexa-, and heptameric IMMADs have no strict constraint with respect to their relative position. Table 6 summarizes the IMMAD occurrence as part of larger sequences and the prominent co-occurring IMMAD partners.

**Table 6:** **Inclusion of IMMADs in larger elements and co-occurring motifs**

Penta-, hexa, and heptameric IMMADs	Larger elements	Co-occurring IMMADs within introns of the same MASS genes
AGCAGAC	CeRep25B minisatellite, 609 base tandem repeats in *frm-1*	GGTCTGC, GATGAC, AGGAG
GGTCTGC	CeRep25B minisatellite	AGCAGAC
CCATCGT	HelitronY4_CE transposon	GATGAC, TCTCTCT, ACYCCA, GAATGTT
GAATGTT	HelitronY4_CE transposon	CCATCGT, TCTCTCT, ACYCCA, CCACA, GATGAC
ACYCCA	ACYCCACA	CCATCGT, TCTCTCT, GATGAC, GAATGTT, CCACA, GTCGT
ACATTCG	-	TCTCTCT
AGCCTCA	AGCCTCAACCAAAA(A)TCTC minisatellite	-
RAGAAG	RAGAAGAAG, AGGAGAAG	AGGAG
WCTTCTT	WCTTCTTCTT	-
GATGAC	-	AGCAGAC, TCTCTCT, CCATCGT, ACYCCA, GAATGTT
TCTCTCT	TCTCTCTCT	GATGAC, CCATCGT, ACATTCG, GTCGT, ACYCCA, GAATGTT
CCAGC	-	TGGAC
TGGAC	-	CCAGC, AGGAG
CAHCC	-	-
GTCGT	GTCGTCGT	TCTCTCT, CCACA, ACYCCA
AGGAG	AGGAGAAG, 609 base tandem repeats in *frm-1*	AGCAGAC, RAGAAG, TGGAC, CCACA
CCACA	-	AGGAG, GTCGT, ACYCCA, GAATGTT

## Discussion

The present study confirmed the hypothesis that specific sequence elements occur more frequently in introns flanking alternate exons in genes where multiple alternative splicing decisions occur, as compared to genes where only one such decision occurs. This observation held even when data were subsampled to correct for potential bias due to systematic differences in gene and intron lengths between the gene groups. Moreover, the two gene groups did not differ in terms of nucleotide composition, chromosomal distribution, and gene product function, which rules out potential confounding effects of those factors.

The results of the comparative analysis of intron sequences between *C. elegans* and *C. briggsae* indicate a large conservation of IMMADs and suggest a conserved function. Indeed, results showed that most IMMADs over-represented in *C. elegans* MASS-SASS gene comparison are also over-represented in the comparison between *C. briggsae* orthologous gene groups*.* One limitation of this analysis, however, is the definition of MASS and SASS genes in *C. briggsae*, which is solely based on sequence conservation and not on data directly addressing the number of alternatively spliced regions among the *C. briggsae* genes. Since more and more data on *C. briggsae* transcriptome are becoming available [36–38], further conservation analyses refining the definition of MASS and SASS genes in *C. briggsae* should become feasible in the future.

The present study shows that the selection of specific intronic elements (IMMADs) is non-random within *C. elegans* MASS genes. First, the usage of specific IMMADs depends on the distance separating the multiple splicing decision sites. This suggests that the presence of specific IMMADs is influenced by structural and/or topological features. Second, IMMADs tend to occur in specific pairs (see summary in Table 6), most of the time with no rigid spacing constraint (minisatellites representing an exception). Taken together, these observations suggest that IMMADs have a specific role in the regulation of multiple alternative splicing decisions along single transcripts. This role might involve specific IMMAD pairs and vary according to gene structure, but its exact nature is unknown at this stage. At least two non-mutually exclusive general mechanisms can be proposed to explain how IMMADs might regulate splicing. First, IMMADs might be recognized by specific regulatory proteins. It was shown here that at least four IMMAD sequences relate to specific binding motifs of human RNA-binding proteins that have homologs in *C. elegans*. Second, IMMADs might be implicated in the creation of RNA secondary structures contributing to regulate alternative splicing [39, 40]. Recent studies have shown that intronic motifs can form long-range secondary structures to affect complex splicing decisions [41–43]. The catalog of candidate elements reported here represents a useful starting point for further studies in *C. elegans.*

## Conclusions

In conclusion, the present findings raise the intriguing possibility that several *cis*-regulatory elements, as well as potential corresponding *trans-*acting factors, are specialized in the regulation of multiple alternative splicing decisions. The present study paves the road for additional research in *C. elegans* aiming at understanding how the different IMMADs work. Furthermore, it will be important to determine whether motifs with identical distributions and potential functions are found in additional species, including human.

## Methods

### MASS and SASS datasets

To generate MASS and SASS gene lists, WormBase release WS235 was used to retrieve all the gene models with more than one transcript isoform. These genes were then categorized as MASS or SASS, based on the analysis of *alternative intron* patterns. Candidate alternative introns were initially defined as introns not present in every transcript isoforms. Next, alternative introns resulting from alternative transcriptional starts were removed from the analysis. Alternative intron positions were then computed to identify alternative introns that overlapped with each other. Overlapping alternative introns are characteristic of alternative 3’ splice sites (A3SS), alternative 5’ splice sites (A5SS), mutually exclusive exon (MXE), and skipped exons (also named cassette exons, CE), which have been considered here as representing single splicing decisions. Genes harboring a single alternative intron or a single set of overlapping alternative introns were defined as SASS. Conversely, genes with two or more non-overlapping alternative introns, or with two or more non-overlapping sets of overlapping alternative introns, were defined as MASS. These situations are illustrated in Figure 1, with the example of mutually exclusive exons. This procedure identified a total of 752 MASS and 1570 SASS genes.

### Identification of IMMADs

Introns flanking alternate exons in the MASS group (3132 sequences) were compared to introns flanking alternate exons in the SASS group (2113 sequences). The Galaxy platform (http://usegalaxy.org/) [44–46] was used for intronic sequence analyses with Compseq [47, 48]. Compseq was used to count the number of occurrences of every possible pentamers (1024), hexamers (4096), and heptamers (16384). For each oligomer, the enrichment in the MASS group as compared to the SASS group was assessed with a Fisher’s exact test, computed in R on the BiostaTGV platform (http://marne.u707.jussieu.fr/biostatgv/). To correct for multiple testing, a conservative Bonferroni approach was applied. The *p*-values reported in Additional files 1 and 2 have been corrected (multiplied by 1024 for pentamers, by 4096 for hexamers, and by 16384 for heptamers).

The significantly enriched oligomeric sequences were grouped according to the following criteria: sequences were clustered in the same group if they were part of each other (e.g. a pentamer being a substring of an hexamer,) or if they diverged by no more than one nucleotide.

### GO term analyses

All GO term analyses were performed with the GOrilla online tool [49, 50].

### MASS and SASS genes stratified subsampling

The MASS and SASS groups of genes diverged in their total length and in the length of the introns flanking alternate exons (see distributions in Figure 2). To get rid of any potential gene length confounding effect when comparing the intronic sequences of MASS and SASS genes, some genes were semi-randomly excluded to re-sample the two groups. Genes were grouped according to their size in bins of 1000 kb. In each bin where the fraction of MASS genes was higher than the fraction of SASS genes, some MASS genes were randomly removed in order to match the fraction observed in the SASS group. The reverse was performed for bins where the fraction of SASS genes was higher than the fraction of MASS genes. The re-sampled groups contained 523 MASS genes (1881 introns flanking alternate exons) and 965 SASS genes (1590 introns flanking alternate exons). This re-sampling also solved the intron length bias.

### Definition and analysis of *C. briggsae* MASS and SASS genes

*C. briggsae* introns of interest were those flanking exons that were orthologous to *C. elegans* alternate exons. The analysis focused on genes with conserved exon sequences and exon-intron structures and for which the definition of orthologous introns is unambiguous. To that end, the lists of *C. elegans* MASS and SASS genes were crossed with a list of genes whose exon-intron structure is conserved across the two species (3404 genes, kindly provided by Juan Fuxman Bass and Marian Walhout) [20]. This method yielded a list of 38 *C. briggsae* MASS genes and a list of 187 *C. briggsae* SASS genes. The sequences of introns flanking exons that are orthologous to alternate exons in *C. elegans* were retrieved and further analyzed to calculate the frequency of specific motifs.

### Comparison with the literature on SREs

For the comparison of the IMMAD list with the pentamers and hexamers reported in Kabat *et al.*[17], the heptamers of the IMMAD list were converted into two hexamers, yielding a total of 79 hexa- or pentamers. Of these, 17 were overlapping with the list of SREs reported in Kabat *et al.* (400 out of 5120 analyzed hexa- or pentamers). This corresponds to an enrichment of 3.24 fold of putative *C. elegans* SREs within the list of IMMADs (*p* = 2.58E-4, by Fisher’s exact test). The comparison with the data from Yeo *et al.*[21] included both upstream and downstream intronic SREs (ISREs).

### Analysis of distances between IMMADs

Distances were calculated between adjacent motifs after extracting the chromosomal coordinates of all the IMMADs found within introns flanking alternate exons.

### Co-occurrence analysis

To calculate the co-occurrence enrichment for a given heterologous A-B motif pair, the number of genes containing (i) A and B, (ii) only A, (iii) only B, and (iv) neither A or B were determined. 2×2 contingency tables were then used to calculate the odds ratios between A-containing and B-containing genes and statistical differences were evaluated by Fisher’s exact tests, with Bonferroni corrections. For any given intron, this analysis only included the motifs whose frequency was higher than expected by chance. This selection process avoided putting an inappropriate weight to large introns, which naturally tend to include more motifs.

### Availability of supporting data

The data sets supporting the results of this article are included within the article (and its additional files).

## Electronic supplementary material

Additional file 1: **63 oligomers identified in the initial MASS-SASS comparison.** This file provides detailed data for each of the 63 oligomers retrieved through the initial IMMAD identification procedure without correcting for gene and intron size. The file includes a summary of motif classification and separate sheets listing pentamers, hexamers, and heptamers significantly enriched in the MASS group versus the SASS group. Fisher’s exact tests were used to assess the oligomer frequency differences between the MASS and SASS groups. Bonferroni corrections were used to compensate for multiple testing and the corrected *p*-values are reported. (XLSX 23 KB)

Additional file 2: **MASS-SASS comparison with size-matched subsamples.** This file contains enrichment data obtained before and after size-subsampling for the 63 relevant oligomers in the MASS/SASS comparison. Fisher’s exact tests were used to assess the oligomer frequency differences between the MASS and SASS groups. Bonferroni corrections were used to compensate for multiple testing and the corrected *p*-values are reported. (XLSX 16 KB)

Additional file 3: **Frequencies of genes harboring specific IMMADs within the MASS and SASS groups.** This spreadsheet presents the numbers and frequencies of genes harboring specific IMMADs within MASS and SASS gene groups. For each IMMAD, a Fisher’s exact test was used to assess the gene frequency difference across the MASS and SASS groups. Bonferroni corrections were used to compensate for multiple testing and the corrected *p*-values are displayed. (XLSX 12 KB)

Additional file 4: **Comparative IMMAD analysis between** ***C. elegans*** **and** ***C. briggsae.*** This file contains the raw data and *p*-values corresponding to Figure 3. Note that values for *C. elegans* are different from those reported in Table 1 and Additional file 2 because only the subsample of MASS and SASS genes with conserved exon-intron structure across *C. elegans* and *C. briggsae* was included in this analysis. (XLSX 12 KB)

Additional file 5: **Full list of GO terms enriched in the MASS group of genes as compared to the whole genome.** This spreadsheet contains the full list of GO terms enriched in the MASS group of genes as compared to the whole genome. (XLSX 20 KB)

Additional file 6: **GO term analyses among MASS genes harboring specific IMMADs.** This spreadsheet contains the lists of GO terms enriched in subsets of MASS genes harboring specific IMMADs. Separate comparisons were made with the whole genome and with alternatively spliced genes (MASS + SASS). (XLSX 14 KB)

## Abbreviations

GO: Gene Ontology
MASS: Multiple Alternative Splicing decision Sites
SASS: Single Alternative Splicing decision Site
IMMADs: Intronic Motifs linked to Multiple Alternative splicing Decisions
SRE: Splicing Regulatory Elements.
